# Effects of Piano Training in Unilateral Cerebral Palsy Using Probabilistic and Deterministic Tractography: A Case Report

**DOI:** 10.3389/fnhum.2021.622082

**Published:** 2021-10-21

**Authors:** Ana Alves-Pinto, Mónica Emch, Renée Lampe

**Affiliations:** ^1^Research Unit of the Buhl-Strohmaier Foundation for Pediatric Neuroorthopaedics and Cerebral Palsy, Orthopaedic Department, Klinikum Rechts der Isar, Technical University of Munich, Munich, Germany; ^2^Department of Neuroradiology, School of Medicine, Klinikum Rechts der Isar, Technical University of Munich, Munich, Germany; ^3^TUM-Neuroimaging Center (TUM-NIC), Technical University of Munich, Munich, Germany; ^4^Graduate School of Systemic Neurosciences, Ludwig-Maximilians-Universität, Munich, Germany; ^5^Markus Würth Stiftungsprofessur, Munich, Germany

**Keywords:** diffusion tensor imaging, fractional anisotropy, sensorimotor, rehabilitation, dorsal cingulate, diffusivity

## Abstract

Cerebral palsy (CP) is an umbrella term encompassing motor and often additional disabilities, resulting from insult to the developing brain and remaining throughout life. Imaging-detected alterations in white matter microstructure affect not only motor but also sensorimotor pathways. In this context, piano training is believed to promote sensorimotor rehabilitation for the multiplicity of skills and neuronal processes it involves and integrates. However, it remains unknown how this contribution may occur. Here, effects of 1.5 years of piano training in an adolescent with unilateral CP were investigated through tests of manual function and by comparing fractional anisotropy, mean diffusivity, radial and axial diffusivity in neuronal pathways pre- vs. post-training. In the absence of a control condition and of data from a larger cohort, both probabilistic neighborhood and deterministic tractography were employed to reduce bias associated with a single-case analysis and/or with user-input. No changes in manual function were detected with the tests performed. In turn, the two tractography methods yielded similar values for all studied metrics. Furthermore, *post-hoc* analyses yielded increased fractional anisotropy accompanied by decreases in mean diffusivity in the bilateral dorsal cingulate that were at least as large as and more consistent than in the bilateral corticospinal tract. This suggests contributions of training to the development of non-motor processes. Reduced anisotropy and correspondingly high mean diffusivity were observed for the bilateral corticospinal tract as well as for the right arcuate and the inferior longitudinal fasciculus, two sensory processing-related pathways, confirming the importance of sensorimotor rehabilitation in CP.

## Introduction

Cerebral palsy (CP) constitutes the most common neurodevelopmental disorder underlying motor impairments occurring during childhood, with a prevalence of 1.5 to 3 cases per 1,000 live births worldwide (Odding et al., 2006; Yeargin-Allsopp et al., 2008). Insult to the brain occurring before, during or shortly after birth affects the development of neuronal regions and pathways, leading to non-progressive, though generally non-static, deficient gross and fine motor abilities. Deficits in motor function have been observed to be associated with alterations in white matter (WM) pathways affecting not only the corticospinal tract but especially sensorimotor pathways (Hoon et al., 2002, 2009; Nagae et al., 2007).

The presence of concomitant disorders, such as different degrees of perceptual and cognitive impairment, difficulties in articulation, behavioral problems and epilepsy (Rosenbaum et al., 2006; Aisen et al., 2011) and altered sensorimotor integration in CP demand rehabilitation strategies that activate and promote the integration of different processes. We hypothesize that music instrument training is an optimal vehicle for this: the repeated performance of movements, the coordination of hands and fingers and its continuous adjustment to the auditory feedback foster the integration of motor, auditory, visual, memory and proprioception processes in the brain.

Music instrument training has been shown to induce neuroplasticity in healthy participants, both at structural and functional level (Schlaug, 2001; Jäncke, 2009; Tervaniemi, 2009; Wan and Schlaug, 2010; Herholz and Zatorre, 2012; Moore et al., 2014). These include changes in WM structure and brain connectivity, both captured with diffusion tensor imaging (DTI; Moore et al., 2014). In clinical populations with motor deficits (e.g., stroke patients) music-instrument training has been observed to contribute not only to improved hand-motor function (Schneider et al., 2007, 2010; Altenmueller et al., 2009; Villeneuve and Lamontagne, 2013) but also to induce neuroplasticty (Rojo et al., 2011; Amengual et al., 2013; Grau-Sánchez et al., 2013).

Patients with CP present however multiple disorders and these since birth. As a consequence, the neurodevelopmental status at the beginning of rehabilitation training is very different to that in other clinical populations. Despite this, a few studies, that focused on the effects of piano training, reported changes in the dynamics of finger key pressing movement and potential changes in connectivity between motor-related areas derived with dynamic causal modeling (Chong et al., 2013; Alves-Pinto et al., 2015, 2017; Lampe et al., 2015). The heterogeneity among patients and in the effects observed demand however further investigation and employing different methodologies.

Here we investigated the effects of piano training in CP by assessing WM neuroplasticity in an adolescent with unilateral CP that learned to play the piano for 18 months. Neuroplasticity was assessed by (1) deriving specific neuronal pathways via tractography, and (2) comparing fractional anisotropy (FA), mean diffusivity (MD), axial diffusivity (AD) and radial diffusivity (RD), biomarkers of WM microstructure, in several neuronal pathways before and after the piano training (Beaulieu, 2002). The pathways analyzed included other tracts beyond those known to be involved in auditory-motor processes, e.g., the arcuate fasciculus (Moore et al., 2017). Given the complexity of the task and of the network-based nature of brain processing it cannot be excluded that neuroplasticity can be induced beyond auditory-motor pathways. By considering a patient with unilateral CP potential differential effects of training on paths ipsilateral and contralateral to the hemisphere affected by the lesion could be observed. Finally two different tractography approaches were employed: (1) probabilistic neighborhood tractography (PNT, Clayden et al., 2006) and (2) deterministic tractography. Since the latter assumes a single orientation at each voxel we also employed PNT, which additionally does not depend on input from the user as do segmentation methods based on regions-of-interest (ROI). If the two approaches, applied independently to the same dataset deliver similar results, then these are less likely to be affected by biases and likely to provide a realistic description of the tracts investigated.

## Case Report and Methods

### Participant

The participant is an adolescent with unilateral spastic CP, left-affected, and who was 16-years old at the beginning of the study. The gross motor function was classified at level 2 according to the Gross Motor Function Classification System (GMFCS; Palisano et al., 1997). Despite spasticity of the left hand, the participant was still able to use it in a limited way, reflected in a Manual Ability Classification System (MACS; Eliasson et al., 2006) level of two. There were no other cases of CP in the family and a genetic origin was excluded.

The participant had no musical training. At the end of the study, the participant reported having enjoyed playing and expressed the wish to continue with the training.

Both the participant and legal guardians were informed about the study and provided their consent to participation before the start of the study. Participation was voluntary. All procedures were in accordance with the Declaration of Helsinki and approved by the Ethics Committee of the Klinikum rechts der Isar of the Technical University of Munich before beginning the study. Data is available on request from the authors.

### Instrumental (Piano) Training

The participant received individualized 30–45-min training sessions of piano with a professional piano teacher, twice a week during a period of 18 months, with breaks during school holidays. An 88-key MIDI controller keyboard (®Casio), was used for the training. Every training session started with about 10 min preliminary exercises consisting in playing, in the pace possible for the participant, a short pentatonic scale moving up and down, beginning with the thumb and ending with the little finger. Exercises were done first with the right hand, then with the left hand and, when possible, with both hands at the same time. Following these exercises, small pieces of folk music were trained.

During the period of training the regular therapy program, consisting of Bobath-based neurological physiotherapy and swimming twice per week, was not interrupted.

### Tests of Manual Function and Piano Tests

The Box & Block (Mathiowetz et al., 1985) and Hand Grip tests were performed with the two hands separately before and after the training (Table 1). The Hand grip test uses a commercial hand dynamometer (Baseline^©^) to measure the strength with which the participant is able to clench the device.

**Table 1:** Changes in the results of the Box & Block, Hand Grip and piano tests with the piano training.

**Box & Block (# cubes)**					
**Left Hand**			**Right Hand**		
before	after	% change	before	after	% change
15	17	13	63	62	−1.6
**Hand Grip (psi)**					
**Left Hand**			**Right Hand**		
before	after	% change	before	after	% change
4.5	1	−77	15	12.5	−16
**Piano Test** **(mean stroke interval in ms from test session 1 to test session 6)**					
28 (session 1) > 31.8 > 35.9 > 33.5 > 35.8 > 34.1 (session 6)					

*For both the Box & Block as well as the Hand Grip tests an improvement in manual ability results in an increase in the number of cubes moved and in the grip force respectively. Only two test sessions were conducted, before and after the training. Piano tests were conducted six times during the training. In this case, an improvement reflects in a decrease in the mean stroke interval*.

A total of six tests at the piano were furthermore conducted at regular intervals during the period of training. They consisted in pressing eight times consecutively a keystroke with a single finger, as regularly as possible, and in repeating this movement with all fingers one after the other. The participant was unable to control differentiated movements with single fingers of the left hand, particularly whilst keeping a rhythmic pace, and therefore no tests were conducted with this hand.

### Acquisition of Diffusion Weighted Images

Diffusion weighted images (DWI) were collected before and after piano training in a 3-Tesla whole body MR scanner (Achieva, Philips, The Netherlands) using an 8-channel phased-array head coil. One T2-weighted (*b* = 0 s mm^−2^) and sets of diffusion weighted (*b* = 1,000 s mm^−2^) single-shot spin-echo echo-planar imaging (EPI) volumes were acquired in the axial plane (*TR* = 14,708 ms, field-of-view *FoV* = 224 x 224 mm^2^, 60 contiguous slices, voxel size = 2 mm^3^) with diffusion gradients applied in 32 non-collinear directions. T1-weighted anatomical images were obtained with a magnetization-prepared rapid acquisition gradient echo (MPRAGE) sequence, with an echo time of 4 ms, a repetition time of 9 ms, inverse time = 100 ms, flip angle = 5°, field of view = 240 x 240 mm^2^, matrix = 240 x 240, 170 slices, voxel size=1mm^3^.

### Preprocessing and Analysis of DWI Images

DWI images were converted from PAR/REC to NifTI-1 format with the dcm2niix function of MRIcro (https://people.cas.sc.edu/rorden/mricro/mricro.html; Rorden and Brett, 2000).

Two different fiber tracking algorithms were employed: (1) PNT, implemented with the software package TractoR (https://www.tractor-mri.org.uk/; Clayden et al., 2011), and (2) deterministic tractography, implemented via ExploreDTI (Leemans et al., 2009).

Visual inspection of data quality did not show the presence of artifacts that would require rejection of data.

#### Preprocessing and Probabilistic Tractography With TractoR

DWI data was preprocessed with the following steps included in the preprocessing pipeline of TractoR: correction of susceptibility-induced distortions and identification of the image with no diffusion weighting with FSL *topup* (Andersson et al., 2003), extraction of the brain volume (Smith, 2002), and correction of eddy current-induced distortions with FSL *eddy_correct* (Andersson and Sotiropoulos, 2016), using as anatomical reference the image volume with no diffusion weighting.

A least-squares fitting algorithm was employed to fit the diffusion tensor and to compute parametric maps of FA, AD, RD and MD. Distributions of diffusion parameters at each voxel were computed using the FSL *bedpostx* function with a two fiber model per voxel (Behrens et al., 2007).

FSL's Bedpost/ProbTrackX algorithm was then employed to perform tract segmentation. For each pathway, B-spline tract representations for a 7 x 7 x 7 neighborhood of voxels around the seed point of the tract of reference (Table 2) were generated (Maniega et al., 2008, 2017), evaluated and matched against the existing model by computing matching (against the pre-trained model) probabilities (Clayden et al., 2007). These are a function of the length and shape of the candidate and reference (or model) tracts. The pre-trained model was the only option given the case-type of study. A mask image was then generated with the best matching model and visualized (Clayden et al., 2009) to make sure the output constituted a reasonable representation of the tract analyzed.

**Table 2:** Comparison of estimated marginal means of FA for the two sessions (session 1: before training, session 2: after training) in the repeated measures model.

**Tract**	**Seed point coordinates (voxels)**	**Session 2-session 1**	***p***	**95% confidence interval**
L arcuate	(161,101,40)	−0.0083	0.6725	[−0.0314, 0.0481]
R arcuate	(84,99,38)	0.0072	0.7158	[−0.0470, 0.0326]
L atr	(141, 156, 29)	0.0204	0.3047	[−0.0601, 0.0194]
R atr	(102, 153, 28)	0.0173	0.3832	[−0.0570, 0.0225]
L dorsal cingulate	(129, 117, 38)	0.0697	**0.0011**	[−0.1095, −0.0300]
R dorsal cingulate	(112, 110, 38)	0.0721	**0.0008**	[−0.1120, −0.0323]
L cst	(146, 114, 32)	0.0253	0.2043	[−0.0651, 0.0145]
R cst	(98, 119, 30)	0.0449	**0.0281**	[−0.0847, −0.0052]
genu	(121,168,30)	0.0089	0.6520	[−0.0486, 0.0309]
L ilf	(169, 103, 24)	−0.0304	0.1289	[−0.0093, 0.0702]
R ilf	(78, 102, 25)	−0.0621	**0.0033**	[0.0223, 0.1018]
splenium	(121, 95,33)	0.0181	0.3596	[−0.0579, 0.0216]
L uncinate	(159, 140, 26)	0.0165	0.4048	[−0.0563, 0.0233]
R uncinate	(93, 142, 22)	0.0140	0.4772	[−0.0538, 0.0257]
L ventral cingulate	(150, 107, 21)	−0.0359	0.0750	[−0.0038, 0.0757]
R ventral cingulate	(104, 108, 22)	0.0676	**0.0015**	[−0.1074, −0.0278]

*The Bonferroni correction was applied to compensate for multiple comparisons (multiple tracts). p values in Bold indicate significant differences. Also indicated are the seed point coordinates for each pathway, for one of the three segmentation runs. Seed point coordinates are based on reference tracts. atr, anterior thalamic radiation; cst, corticospinal tract; ilf, inferior longitudinal fasciculus; R, right hemisphere; L, left hemisphere*.

Model-tract segmentation was performed for the 16 tracts (Table 2) for which there are pre-trained models in the version v3.3.0 (distribution v3.3.1) of the TractoR package used (Maniega et al., 2008). Therefore the method does not depend on the definition of preexisting ROIs.

##### Statistical Analysis

Reported below are the average diffusion and anisotropy values weighted by the probability of connection to the seed point and computed along the best-matching tract. These weighted averages were computed with statistical functions of TractoR for each of the 16 segmented tracts and from DTI data collected before and after the training. Due to its probabilistic nature the PNT algorithm delivered, for each segmented tract, a slightly different model. To account for the variability inherent to the tracts and associated weighted averages segmentation was repeated three times for each tract, such that three weighted average values of FA, MD, AD and RD per track could be sampled.

A one-way repeated measures ANOVA was then employed to evaluate changes in weighted-average FA values between imaging sessions (with pre- and post-training as the two levels for the within-subjects factor “Session” in the ANOVA) over all tracts. A multiple comparison of the weighted averages, Bonferroni-corrected for multiple comparisons, was performed *post-hoc* to assess for which tracts changes were significant. Statistical analyses were performed with the statistics toolbox of MATLAB 2019a.

#### Preprocessing and Deterministic Tractography With ExploreDTI

Subject motion, eddy current and echo planar imaging distortions were corrected using ExploreDTI, version v4.8.6 (Leemans et al., 2009). Images collected in Session 2 were additionally registered to the anatomical T1 image of the Session 1, such that tractography and pathway extraction could be performed on a common native space. The deterministic streamline approach was employed to perform whole brain tractography (Basser et al., 2000), with the default parameters: seed fractional threshold of 0.2; angle threshold of 30°, fiber length of 50 to 500-mm, seed point uniform resolution of 2-mm, no random perturbation of seed points.

The same 16 fiber tracts analyzed with TractoR were then extracted in ExploreDTI from ROIs defined manually for each of the tracts. By performing whole brain tractography before tract extraction the influence of user-bias on the definition of the ROI was reduced. Since tractography was conducted in the same space, the same ROIS defined manually for Session 1 were then used to extract pathways for Session 2. All tracts were visually compared with published ones, derived from healthy adults, before calculating measures of interest within the tracts. Reported below are the median values of FA, MD, AD and RD and associated 95% confidence intervals computed along each of the tracts. Median values were considered to reduce eventual effects of outliers. No statistical tests were performed with these measures.

## Results

No improvements were detected by any of the performed tests of manual function (Table 1). In particular, a reduction of the mean keystroke interval, that would indicate a more regular way of pressing the keys, was not observed.

**Figure 1:**
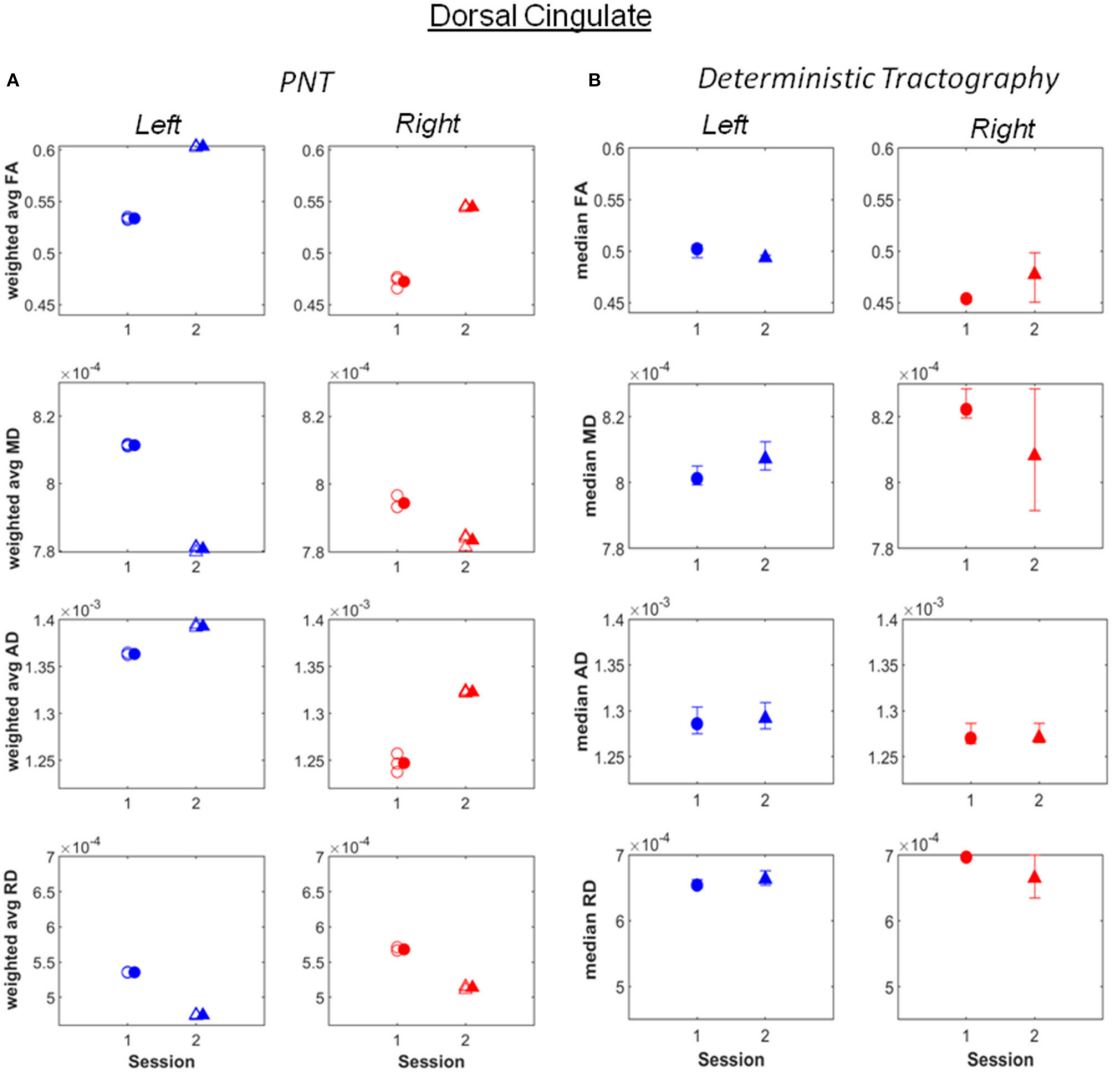
**(A)** Weighted average values of FA, MD, AD and RD computed along the best matching (relative to reference model tract) dorsal cingulate tract, before (session 1) and after (session 2) the piano training. **(B)** Median values of FA, MD, AD and RD computed along the tract, also for before (session 1) and after (session 2) the training. Error bars illustrate 95% confidence intervals. L, left hemisphere; R, right hemisphere.

**Figure 2:**
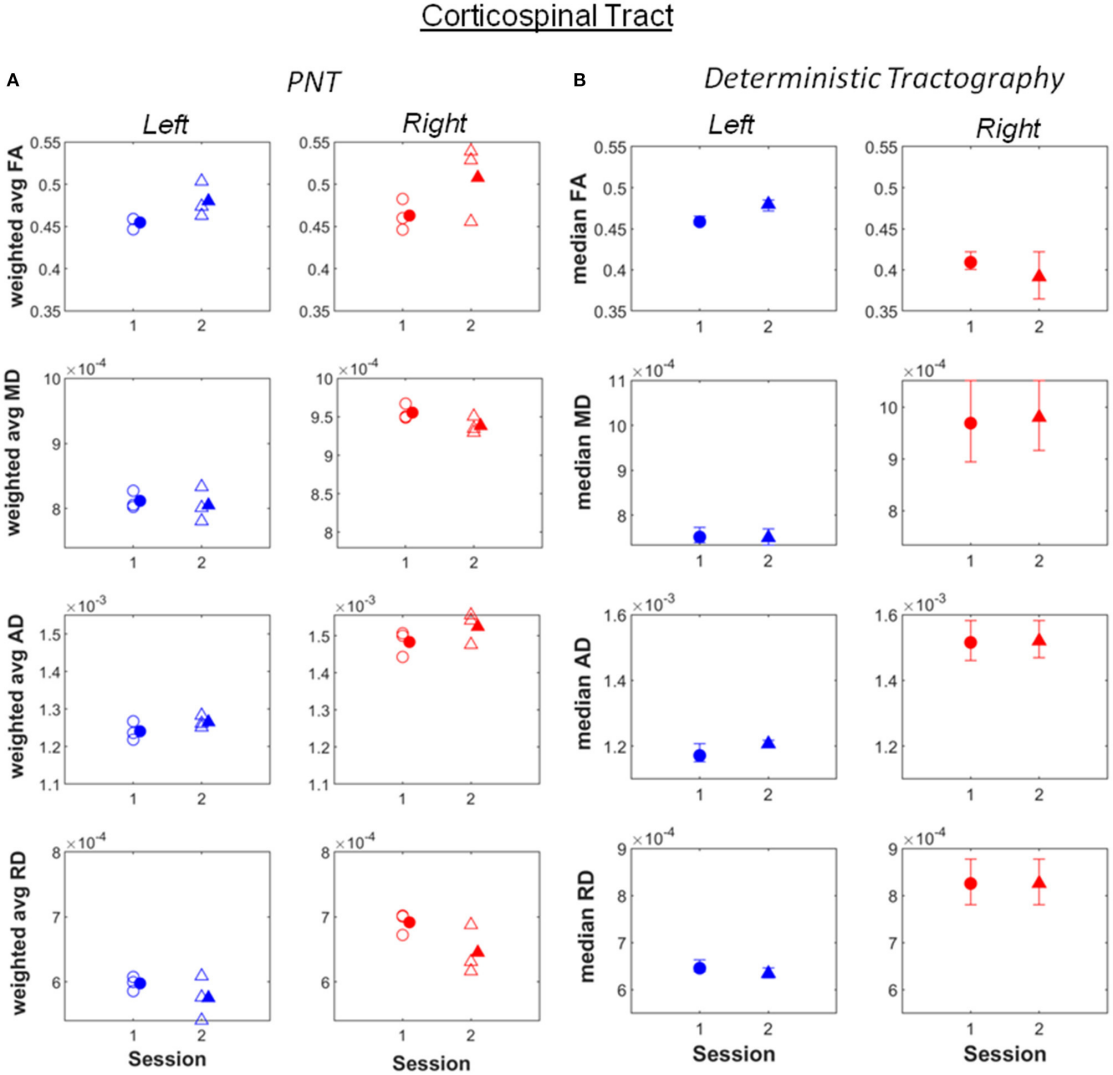
**(A)** Weighted average values of FA, MD, AD and RD computed along the best matching (relative to reference model tract) corticospinal tract, before (session 1) and after (session 2) the piano training. **(B)** Median values of FA, MD, AD and RD computed along the tract, also for before (session 1) and after (session 2) the training. Error bars illustrate 95% confidence intervals. L, left hemisphere; R, right hemisphere.

Increases in weighted average FA and AD computed after PNT, accompanied by decreases in MD and RD, were observed for the bilateral dorsal (Figure 1; see also Supplementary Material), right ventral cingulate tracts (see Supplementary Material) and for the bilateral corticospinal tract (CST; Figure 2; Supplementary Material). Slight increases in FA, though in some cases without associated decreases in MD and RD, were obtained for the right arcuate, anterior thalamic radiation, uncinate, genu and splenium (see Supplementary Material). Decreases in average weighted FA were obtained for the bilateral inferior longitudinal fasciculus, the left ventral cingulate and left arcuate. A one-way repeated measures ANOVA performed on the weighted average values of FA estimated with the PNT-approach showed a significant effect of Session (*F*_(1, 32)_ = 9.86, *p* = 0.0036) as well as a significant interaction between the factors Session and Tract (*F*_(15, 32)_ = 3.73, *p* = 0.00087). A *post-hoc* multiple comparison test showed significant differences for the bilateral dorsal cingulate, the right CST, right inferior longitudinal fasciculus and the right ventral cingulate (Table 2).

Most of the changes in FA, MD, AD and RD observed after PNT were also obtained with deterministic tractography, not only in terms of the absolute values but also in the direction of change. Exceptions were observed for the right inferior longitudinal fasciculus, the right corticospinal tract, the left dorsal cingulated and genu (Supplementary Material).

To quantify the concordance between PNT and deterministic tractography we conducted Kendall's tau correlation test on weighted average FA and median FA values (*r* = 0.48 for first run of PNT, *r* = 0.51 for the second, *r* = 0.44 for third). The positive correlation suggests agreement between the two methods.

Remarkable were also differences in absolute values of FA, MD, AD and RD between the right and left hemispheres obtained for the inferior longitudinal fasciculus, the arcuate fasciculus and uncinate tracts. In comparison, the right and left-CST, one of the most relevant tracts in motor processing, showed similar values for FA, MD, AD and RD (Figure 2).

## Discussion

Both PNT and deterministic tractography yielded comparable measures of diffusion and anisotropy for all tracts analyzed. Although the original images were the same, both the preprocessing and tractography algorithms were different for the two methods. The agreement observed supports the reliability of the results obtained. Given the user-independent approach of fiber tracking in PNT, this agreement suggests an unlikely influence of researcher-based bias.

It should be noticed that the FA and MD values reported are, for several tracts, within the ranges reported to reflect a normal WM structure, namely FA values varying approximately between 0.5 and 0.8 and MD values varying about 600 and 1200 x 10^−6^ mm^2^/s (Moore et al., 2014). In particular, the values of MD estimated for the bilateral CST (Figure 2) are also within these ranges, the values of FA being slightly lower but similar between hemispheres. Whilst it cannot be excluded that tractography was unable to segment areas with pathology and retrieved “artificially” model-like ones, the above observations may also indicate that motor impairments in this patient likely reflect damage to other tracts besides motor pathways, namely sensory and sensorimotor tracts (Hoon et al., 2002). Indeed, particularly low FA values, correspondingly accompanied by high MD values, were observed for the right inferior longitudinal fasciculus and the right arcuate. The first is a long-range tract connecting occipital and temporal areas to anterior temporal areas, involved in processing visual information and visually-guided behaviors (Herbet et al., 2018). The arcuate fasciculus in turn, connects auditory and motor areas (Schlaug et al., 2011; Zipse et al., 2012). This tract is, in this patient, very close to the lesion. The correct processing and integration of visual information and of auditory feedback resulting from self-produced movements is essential for its correct performance. This observation adds therefore to the increasing evidence for the need of therapies promoting sensorimotor integration, supported on imaging (e.g., DTI)-based information about the neuronal pathways and processes affected in each individual, not only in CP but also for instance in stroke patients (Borich et al., 2015).

The increase in FA pre- vs. post-training was significant for the right dorsal cingulate and at least as large as that observed for the right CST, and more consistent across runs. Changes in the left dorsal cingulated were only detected with PNT. The dorsal cingulate connects frontal, parietal and medial temporal areas, interconnecting areas of the limbic system. It is an heterogeneous and long tract that is involved in several processes, namely emotion, reward, pain and motor processes (Bubb et al., 2018). Hence, despite not being a tract directly involved in motor processes, it can affect these in several ways, through the different neuronal mechanisms it supports. The changes in FA observed in the bilateral CST were less consistent across runs relative to the dorsal cingulated, with both methods yielding similar values and changes for the different metrics between sessions, with the exception of FA for the right CST. The significant changes in FA obtained for the right ventral cingulate and right inferior longitudinal fasciculus are not consistent between the two tractography methods. This could mean that these results are less reliable.

The increase in FA in the dorsal cingulate cannot be exclusively attributed to the piano training based on the results presented. First, changes may partially reflect growth-associated processes: The patient was 16 years old at the beginning of the study and the microstructure of neuronal pathways is known to continue developing throughout adulthood (Lebel and Beaulieu, 2011; Lebel et al., 2012). Changes may additionally have resulted from the normal therapy that was continued during the period of piano training. In fact, no parallel improvements in manual function were captured in this patient by the piano, Box & Block or hand grip tests. Nevertheless, given the magnitude of change in this relative to other tracts and relative to reported changes in FA with age in adolescence (e.g., in Lebel and Beaulieu, 2011), and considering the role of the dorsal cingulate in multiple neuronal processes, it is feasible that the effects reported are at least partly due to piano training. Changes in FA have been associated with motor skill learning also in stroke patients (Borich et al., 2014) and motor learning has been suggested to underlie music supported training effects (Grau-Sánchez et al., 2020). And though behavioral measures of manual function did not change, it is possible that other processes, also emotional processes, may have been triggered (Haslbeck et al., 2020). The fact that changes in the dorsal cingulate were similar for both hemispheres suggests that training-associated effects are not related with hemisphere-bound affected neuronal processes in this patient.

It may also be argued that the differences presented may result from tractography having been performed at different time points. Had this however been the case, differences in diffusion parameters would have likely been found for a larger number of tracts than here reported, also for tracts unrelated to sensorimotor processes. Though such time-related effects may still be present, they do not fully explain the results reported.

Given the multiplicity of skills and processes involved in playing piano and the wide range of neuronal alterations that can occur in CP, additional and/or different effects of piano training than the ones reported for this clinical case are likely in other patients. Studies considering longer periods of training, a large group of patients, a control condition or group will likely reveal other effects. Overall the observations reported here support and demand further investigation of training-related effects on WM pathways in CP by means of DTI-based tractography.

## Data Availability Statement

The datasets presented in this article are not readily available because of the personal character of the content. Requests to access the datasets should be directed to renee.lampe@tum.de.

## Ethics Statement

The studies involving human participants were reviewed and approved by Ethics Committee of the Klinikum Rechts der Isar, Technical University of Munich. Written informed consent to participate in this study was provided by the participants' legal guardian/next of kin.

## Author Contributions

AAP and ME contributed to the analysis of the data and to the writing of the manuscript. RL designed the study, collected the data, contributed to the analysis of data, and writing of the manuscript. All authors contributed to the article and approved the submitted version.

## Funding

Research financed by the Buhl-Strohmaier Foundation and the Würth Foundation.

## Conflict of Interest

The authors declare that the research was conducted in the absence of any commercial or financial relationships that could be construed as a potential conflict of interest.

## Publisher's Note

All claims expressed in this article are solely those of the authors and do not necessarily represent those of their affiliated organizations, or those of the publisher, the editors and the reviewers. Any product that may be evaluated in this article, or claim that may be made by its manufacturer, is not guaranteed or endorsed by the publisher.
