# Supplementary material for: Effects of Piano Training in Unilateral Cerebral Palsy Using Probabilistic and Deterministic Tractography: A Case Report
Source: Front Hum Neurosci. 2021 Oct 21;15:622082. doi: 10.3389/fnhum.2021.622082 (PMC8567167; doi:10.3389/fnhum.2021.622082)

Upper panels: Best matching right and left dorsal cingulate tracts segmented with PNT. Both tracts reconstructed from imaging before (session 1) and after (session 2) the piano training are presented. It should be noticed that, due to the probabilistic nature of the PNT method, the best matching tract differs slightly between runs. Lower panels: left dorsal cingulate tracts reconstructed with deterministic tractography, also for before and after the training.

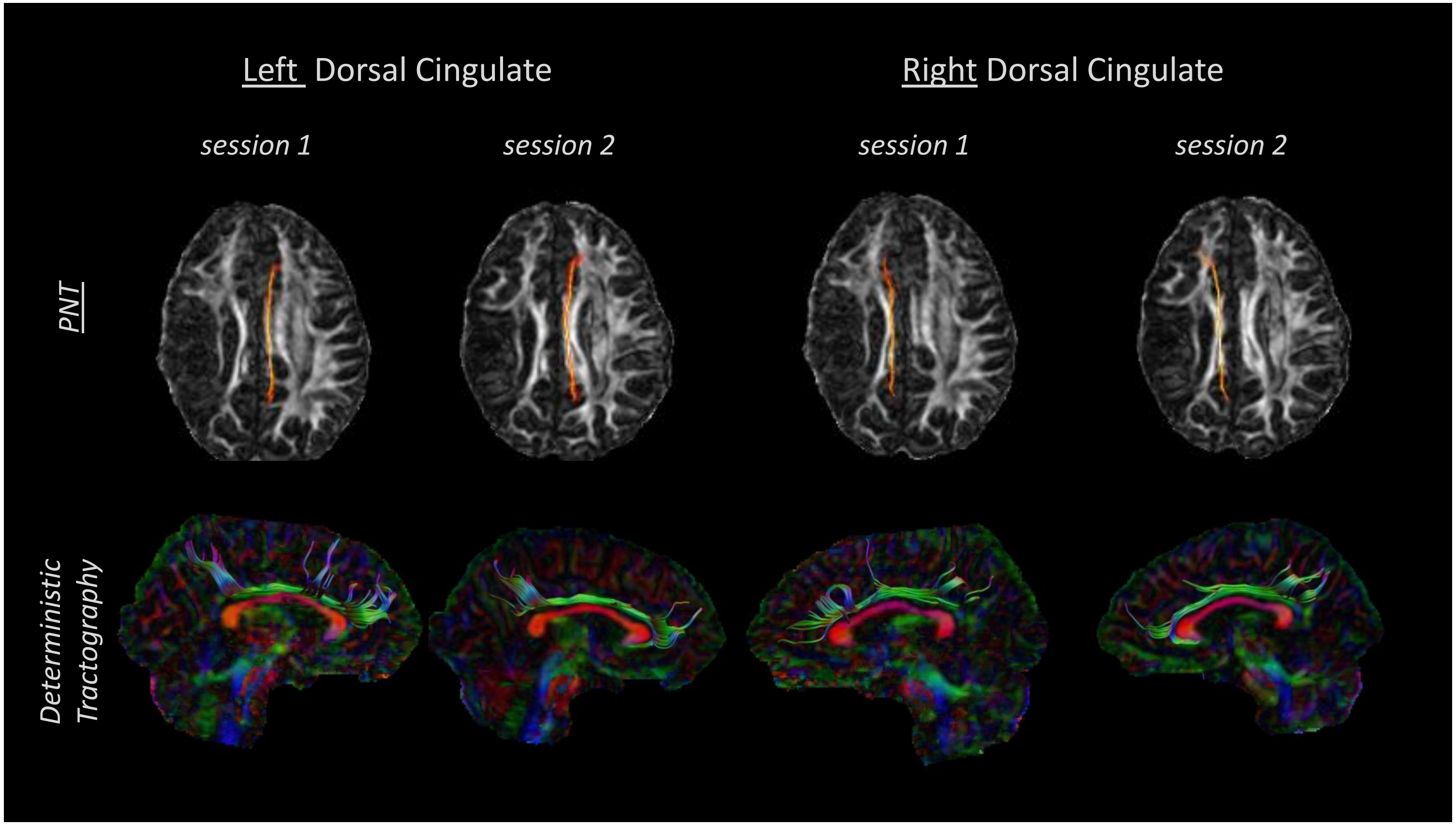

Upper panels: Best matching right and left corticospinal tracts segmented with PNT. Both tracts reconstructed from imaging before (session 1) and after (session 2) the piano training are presented. Lower panels: left and right corticospinal tracts reconstructed with deterministic tractography, also for before and after the training.

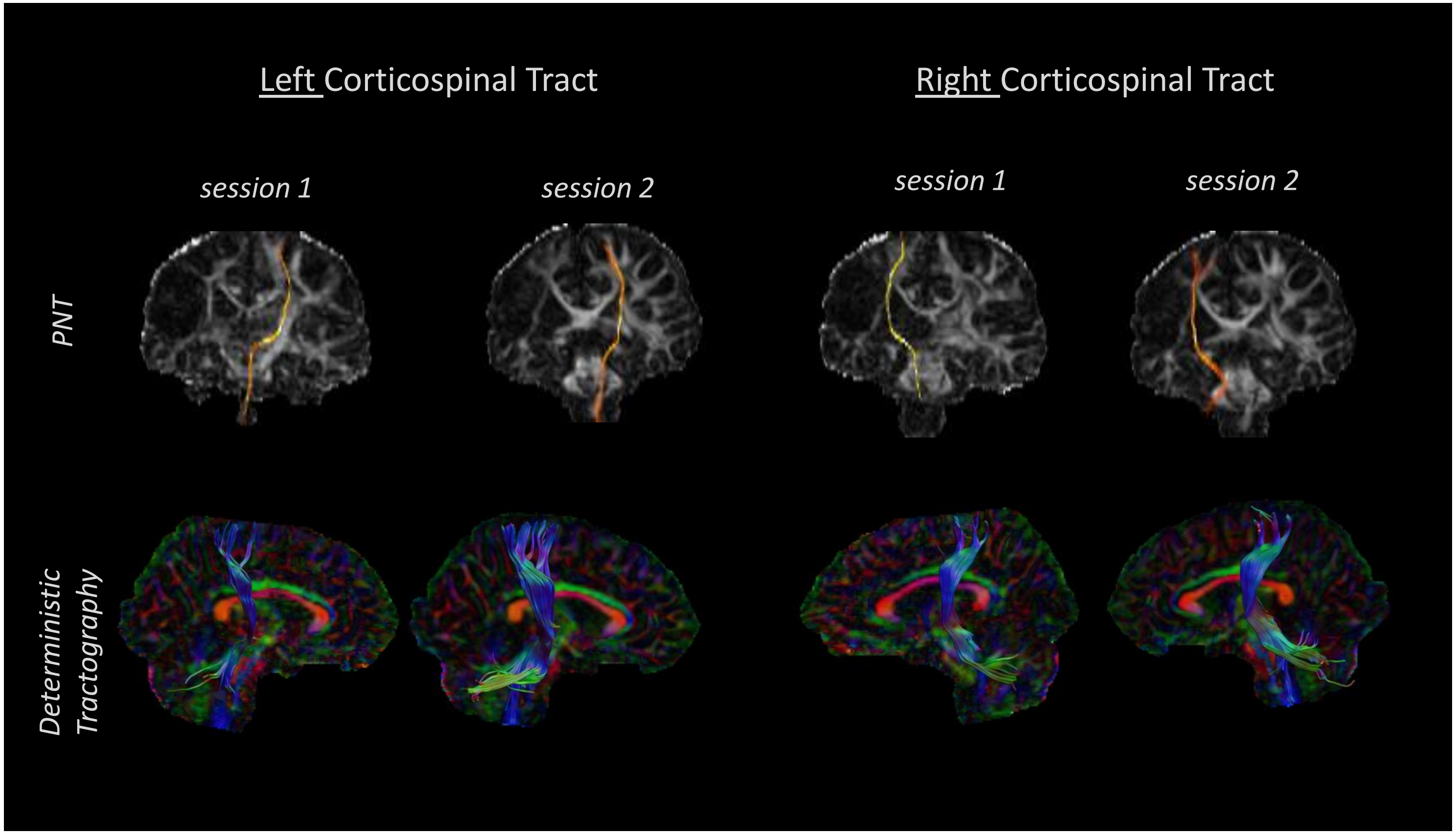

Upper panels: Best matching right and left inferior longitudinal fasciculus segmented with PNT. Both tracts reconstructed from imaging before (session 1) and after (session 2) the piano training are presented. Lower panels: left and right inferior longitudinal fasciculus reconstructed with deterministic tractography, also for before and after the training.

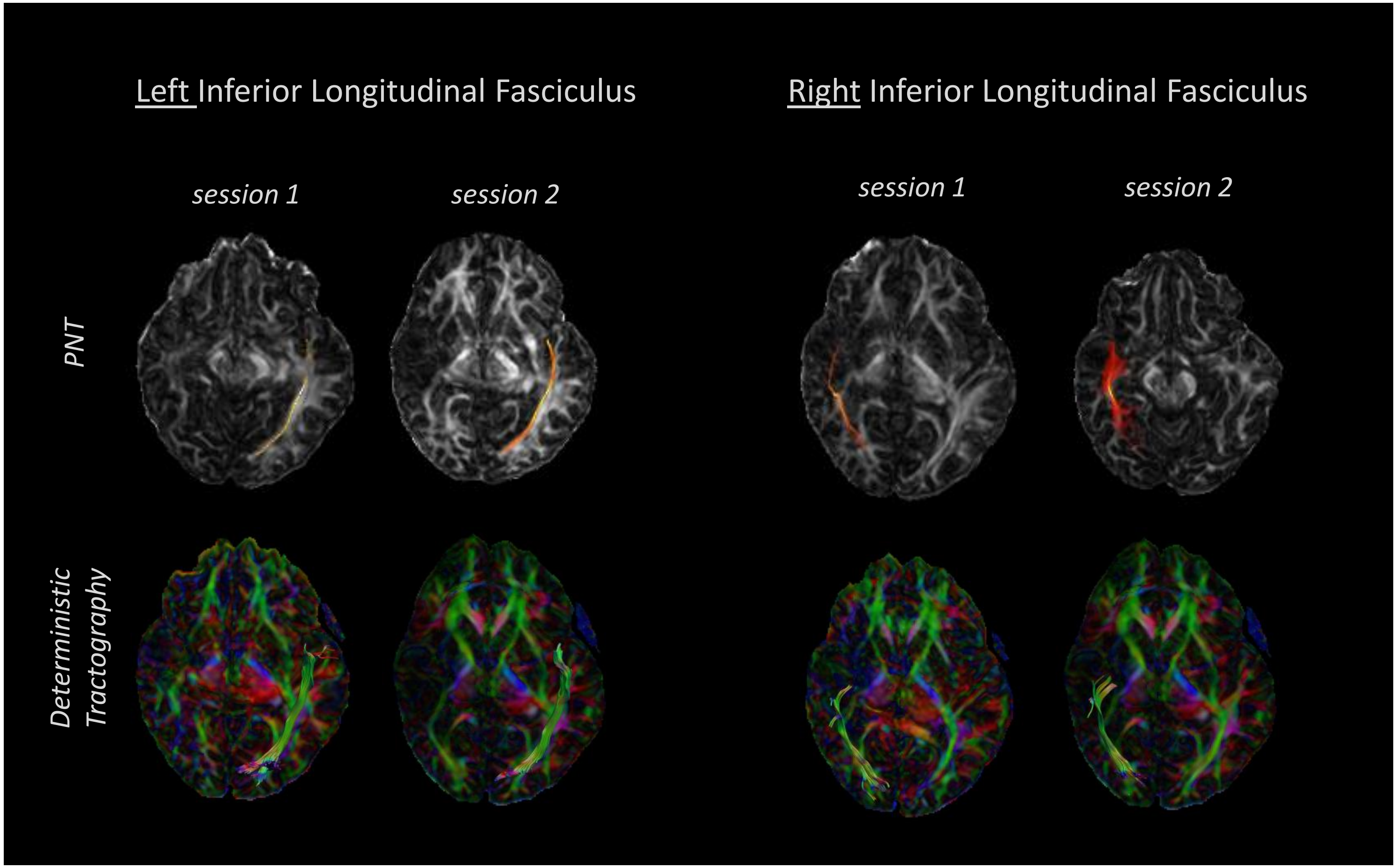

(a) Weighted average values of FA, MD, AD and RD, computed along the best matching (relative to reference model) inferior longitudinal fasciculus tract determined with PNT. Values determined from tracts reconstructed from imaging data collected before (session 1) and after (session 2) the piano training are presented. **(b)** Median values of FA, MD, AD and RD computed along the inferior longitudinal tract derived with deterministic tractography, before (session 1) and after (session 2) the training. Error bars illustrate 95% confidence intervals. L: left hemisphere; R: Right hemisphere.

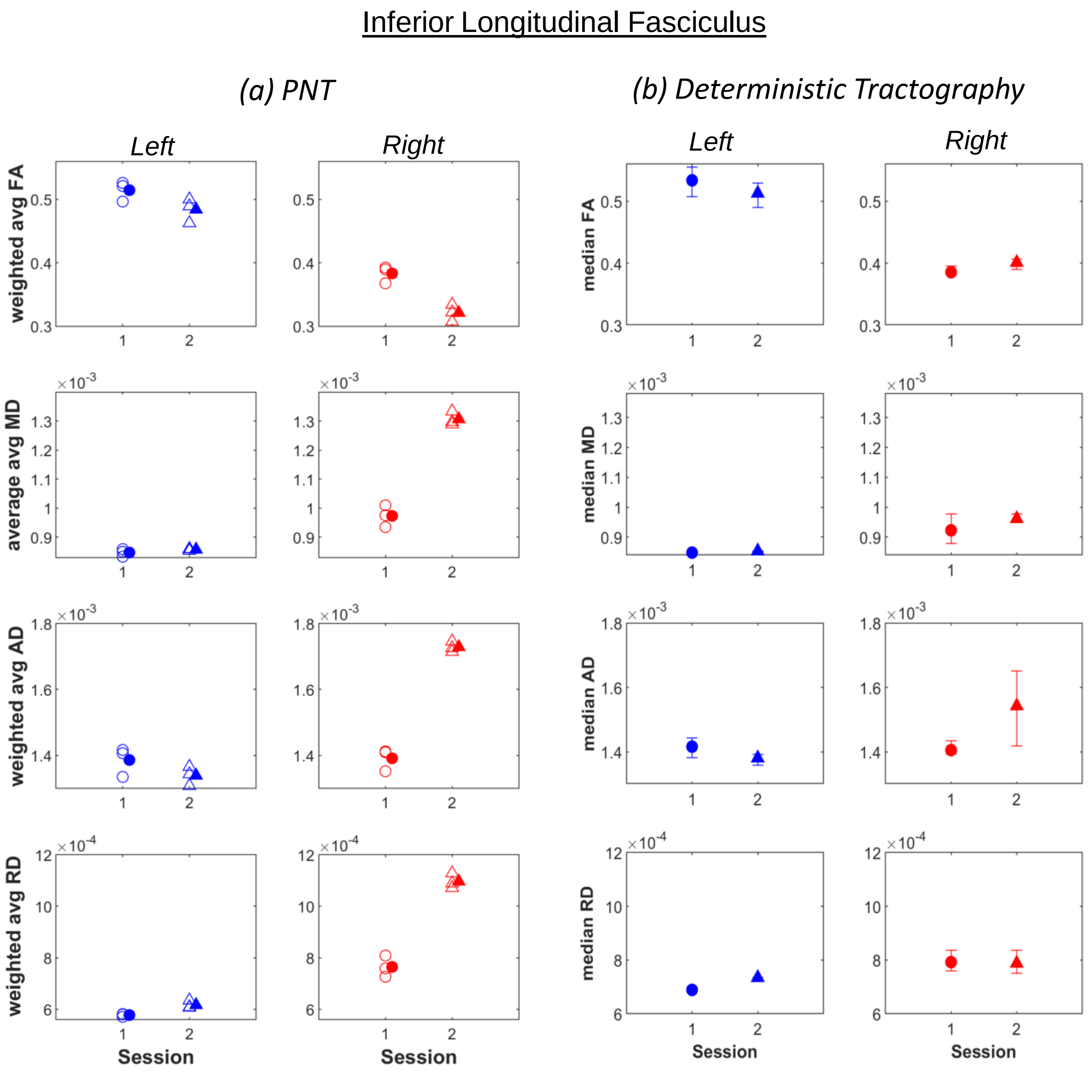

Upper panels: Best matching right and left ventral cingulate tracts segmented with PNT. Both tracts reconstructed from imaging before (session 1) and after (session 2) the piano training are presented. Lower panels: left and right ventral cingulate tracts reconstructed with deterministic tractography, also for before and after the training.

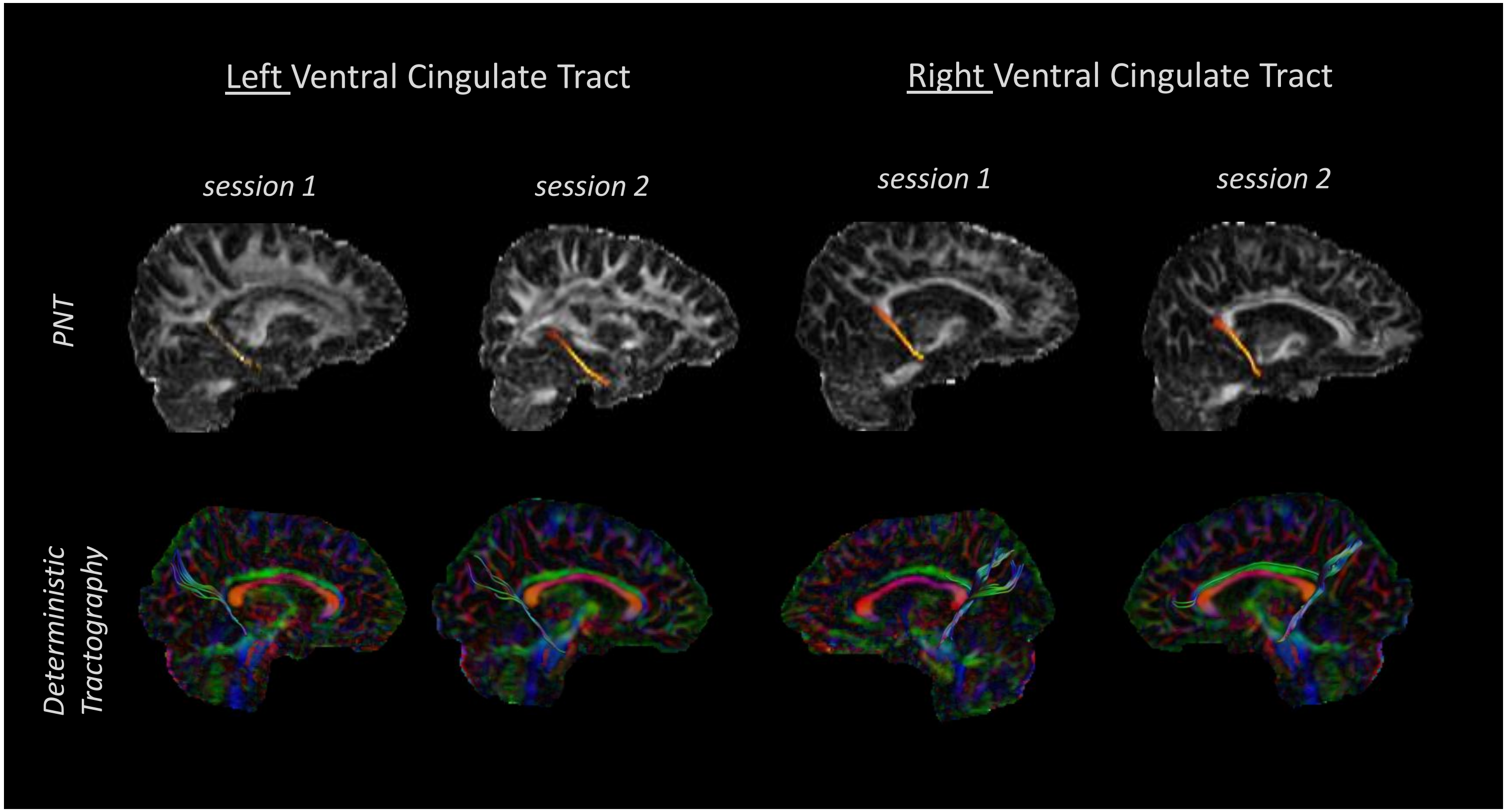

(a) Weighted average values of FA, MD, AD and RD, computed along the best matching (relative to reference model) ventral cingulate tracts determined with PNT. Values determined from tracts reconstructed from imaging data collected before (session 1) and after (session 2) the piano training are presented. **(b)** Median values of FA, MD, AD and RD computed along the ventral cingulate tracts derived with deterministic tractography, before (session 1) and after (session 2) the training. Error bars illustrate 95% confidence intervals. L: left hemisphere; R: Right hemisphere.

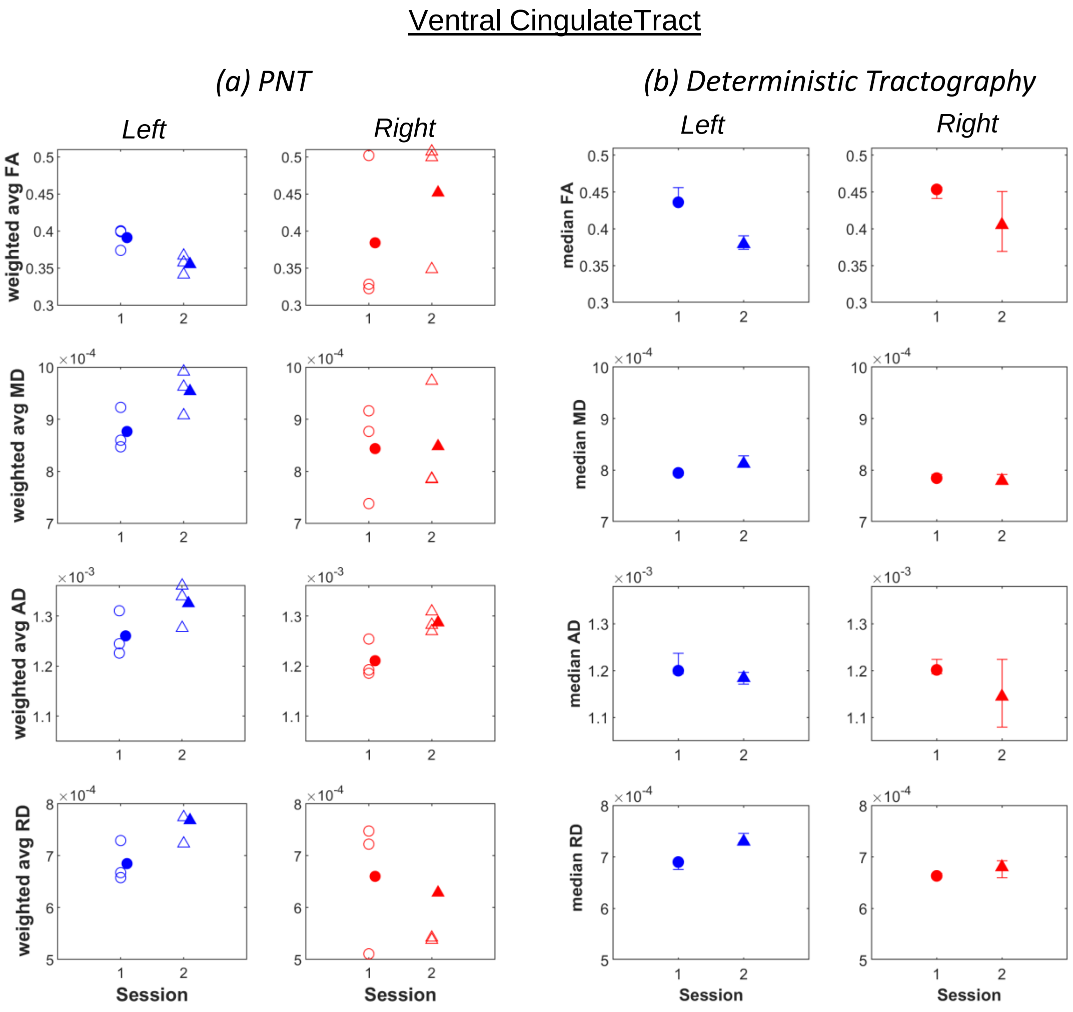

Upper panels: Best matching right and left arcuate tracts segmented with PNT. Both tracts reconstructed from imaging before (session 1) and after (session 2) the piano training are presented. Lower panels: left arcuate tract reconstructed with deterministic tractography, also for before and after the training. The right tract could not be reconstructed based on manual definition of ROIs.

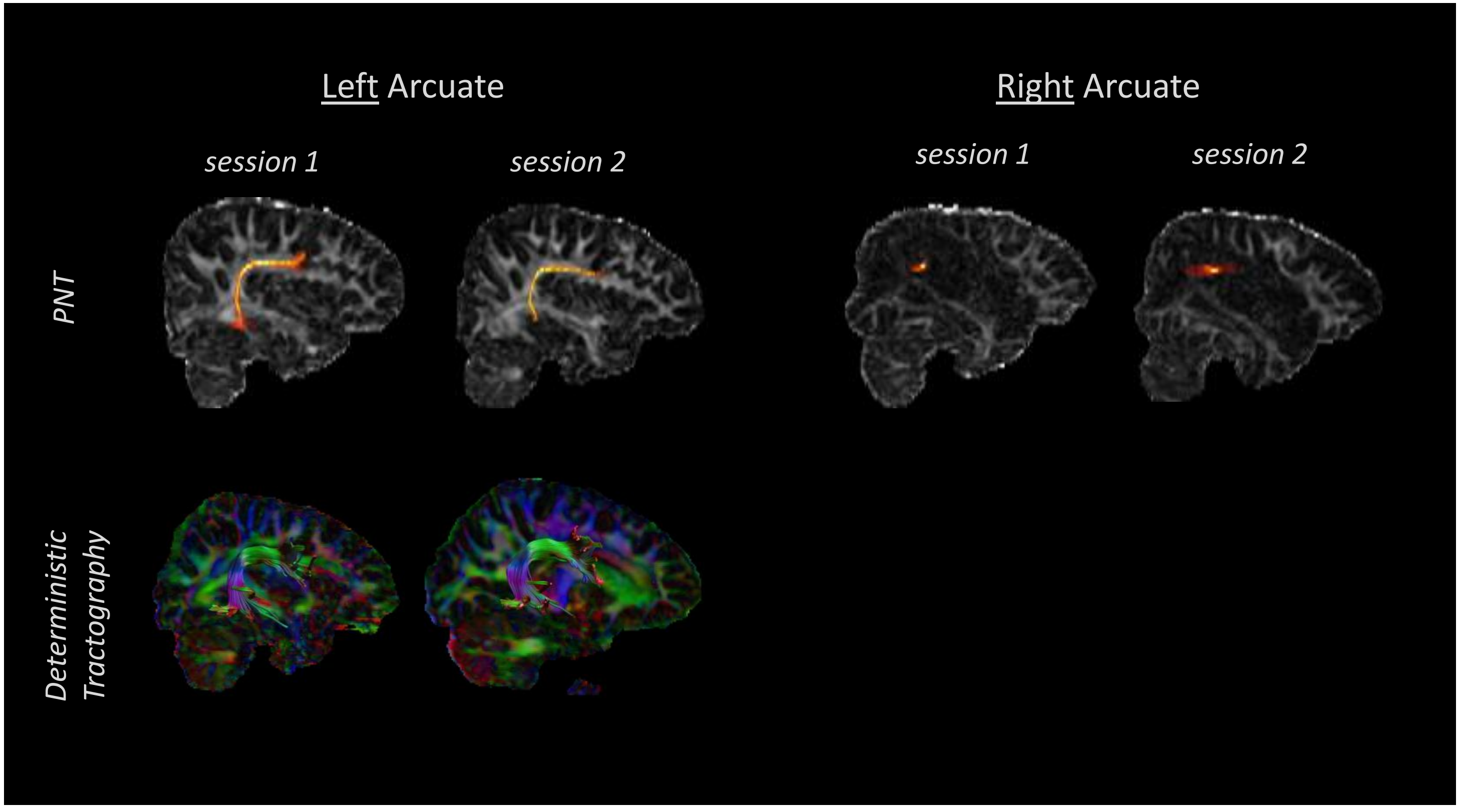

(a) Weighted average values of FA, MD, AD and RD, computed along the best matching (relative to reference model) arcuate tracts determined with PNT. Values determined from tracts reconstructed from imaging data collected before (session 1) and after (session 2) the piano training are presented. **(b)** Median values of FA, MD, AD and RD computed along the left arcuate tract derived with deterministic tractography, before (session 1) and after (session 2) the training. The right tract could not be reconstructed based on manual definition of ROIs. Error bars illustrate 95% confidence intervals. L: left hemisphere; R: Right hemisphere.

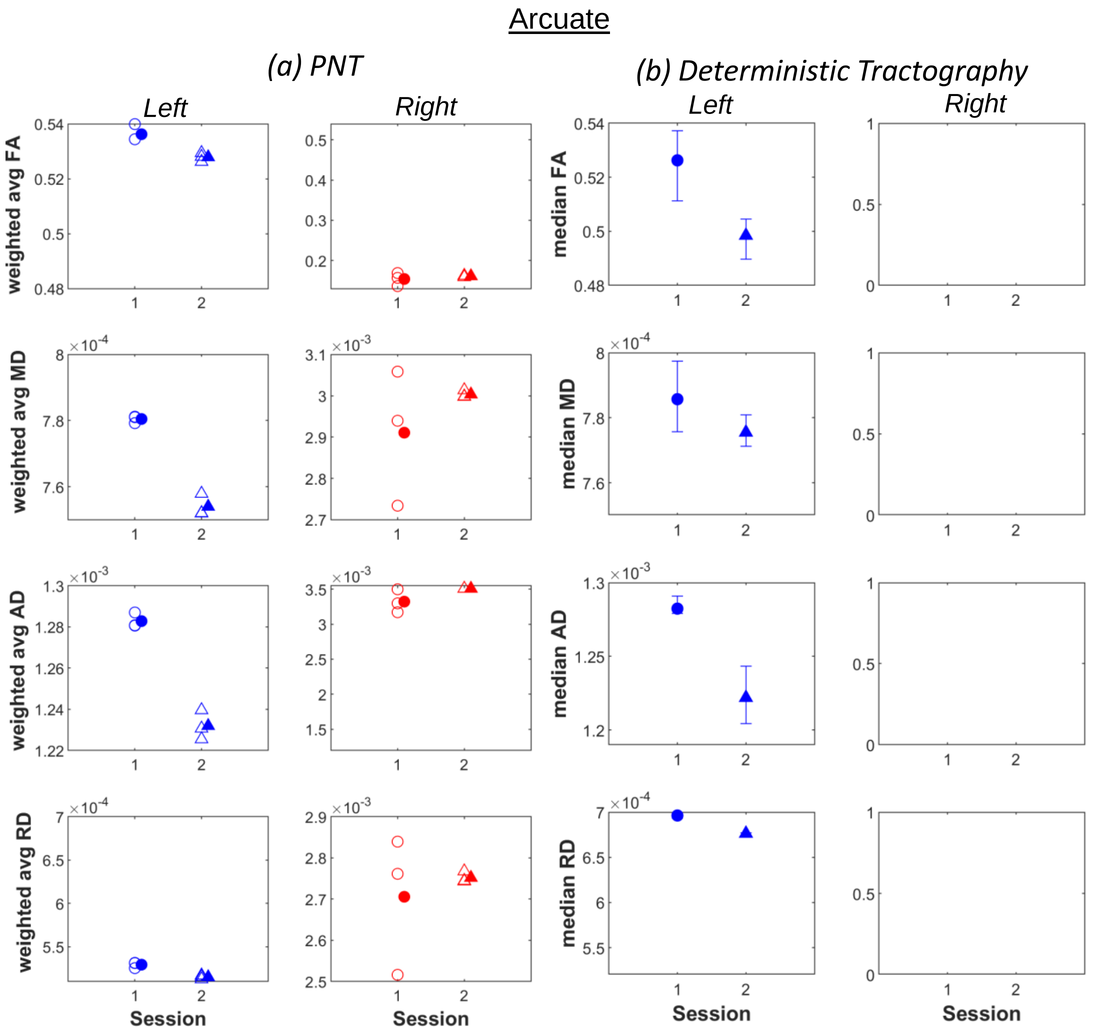

Upper panels: Best matching right and left anterior thalamic radiation segmented with PNT. Both tracts reconstructed from imaging before (session 1) and after (session 2) the piano training are presented. Lower panels: bilateral anterior thalamic radiation reconstructed with deterministic tractography, also for before and after the training.

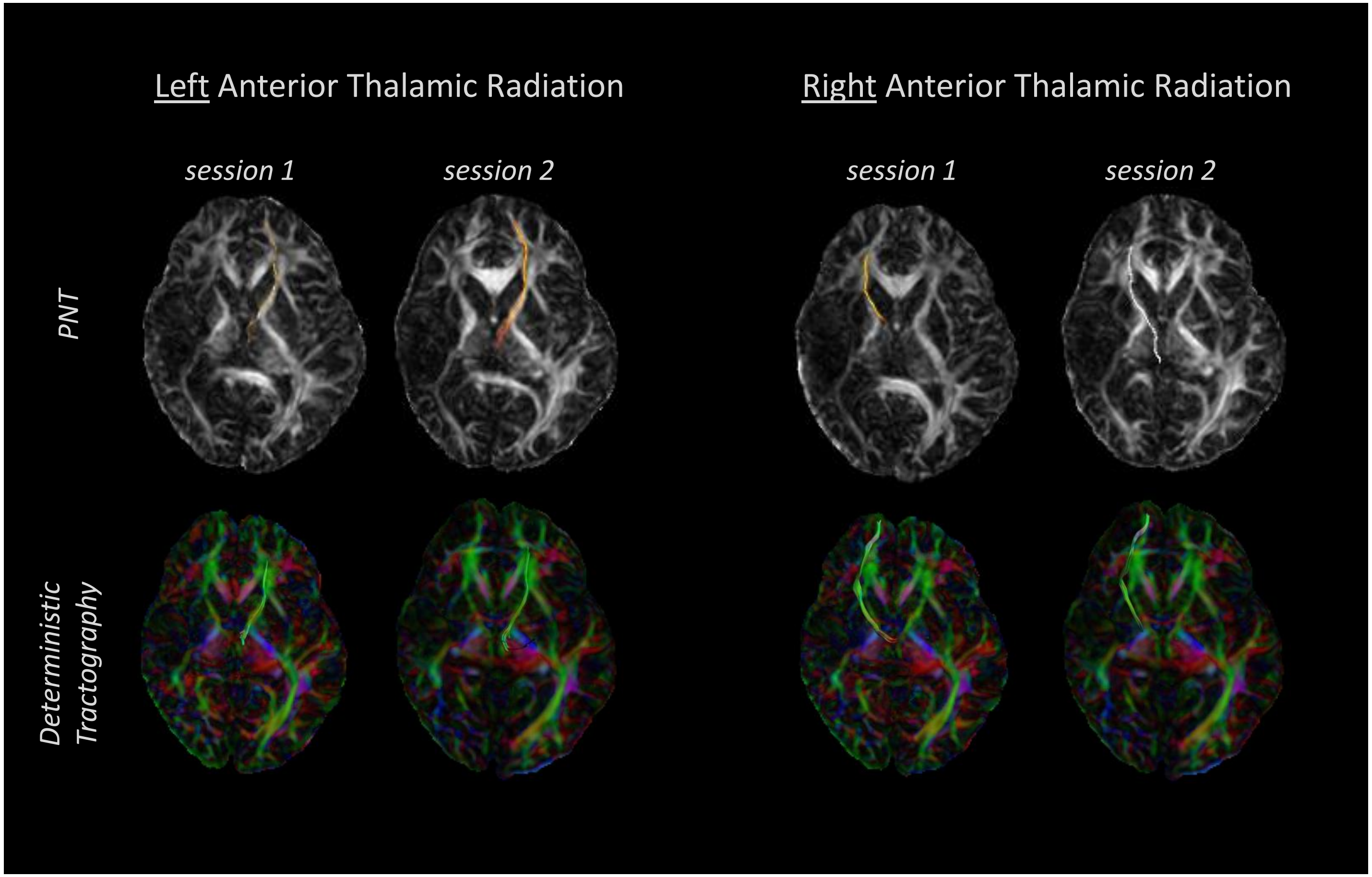

(a) Weighted average values of FA, MD, AD and RD, computed along the best matching (relative to reference model) anterior thalamic radiation determined with PNT. Values determined from tracts reconstructed from imaging data collected before (session 1) and after (session 2) the piano training are presented. **(b)** Median values of FA, MD, AD and RD computed along the bilateral anterior thalamic radiation derived with deterministic tractography, before (session 1) and after (session 2) the training. Error bars illustrate 95% confidence intervals. L: left hemisphere; R: Right hemisphere.

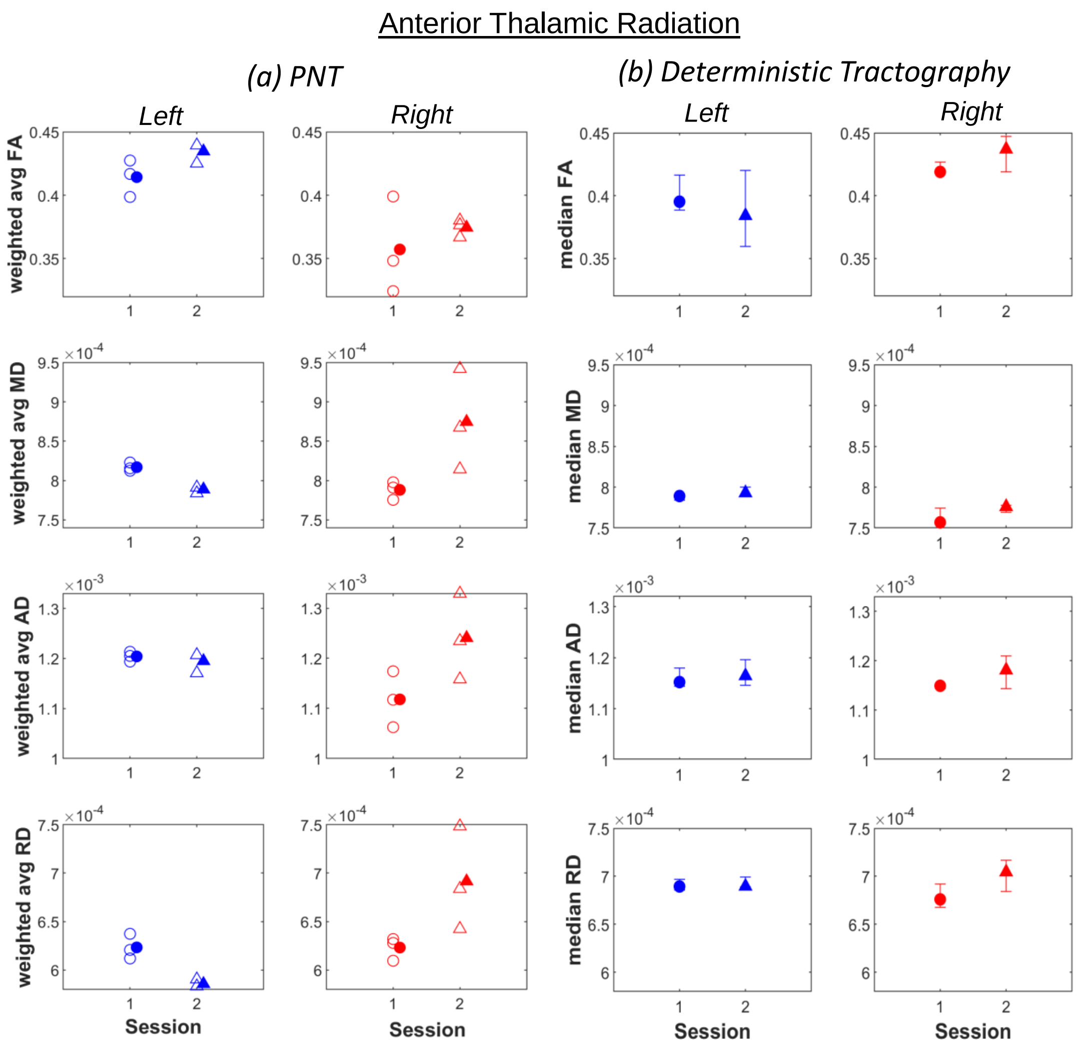

Upper panels: Best matching right and left uncinate tracts segmented with PNT. Both tracts reconstructed from imaging before (session 1) and after (session 2) the piano training are presented. Lower panels: bilateral uncinate tract reconstructed with deterministic tractography, also for before and after the training.

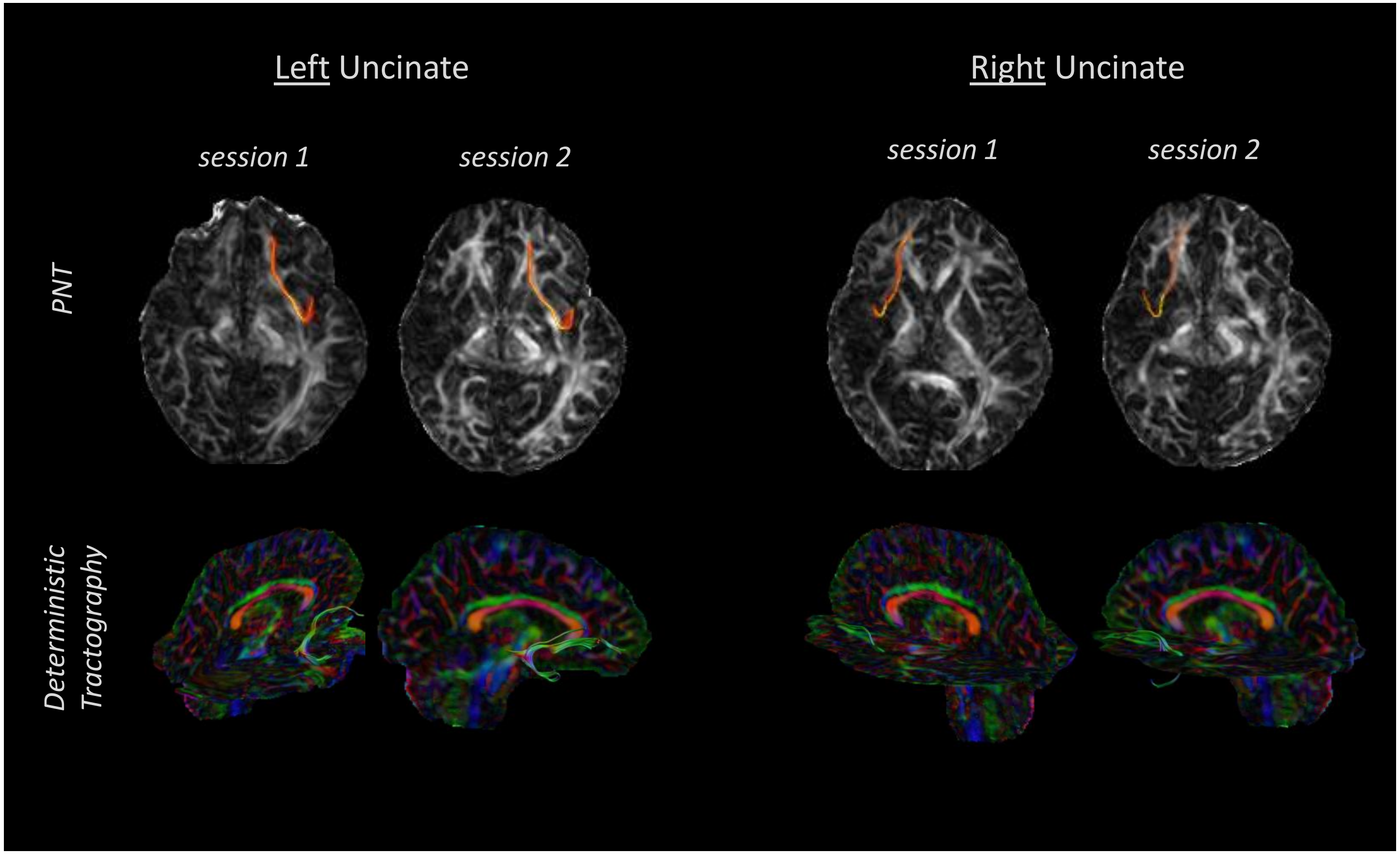

(a) Weighted average values of FA, MD, AD and RD, computed along the best matching (relative to reference model) uncinate tract determined with PNT. Values determined from tracts reconstructed from imaging data collected before (session 1) and after (session 2) the piano training are presented. **(b)** Median values of FA, MD, AD and RD computed along the bilateral uncinate tracts derived with deterministic tractography, before (session 1) and after (session 2) the training. Error bars illustrate 95% confidence intervals. L: left hemisphere; R: Right hemisphere.

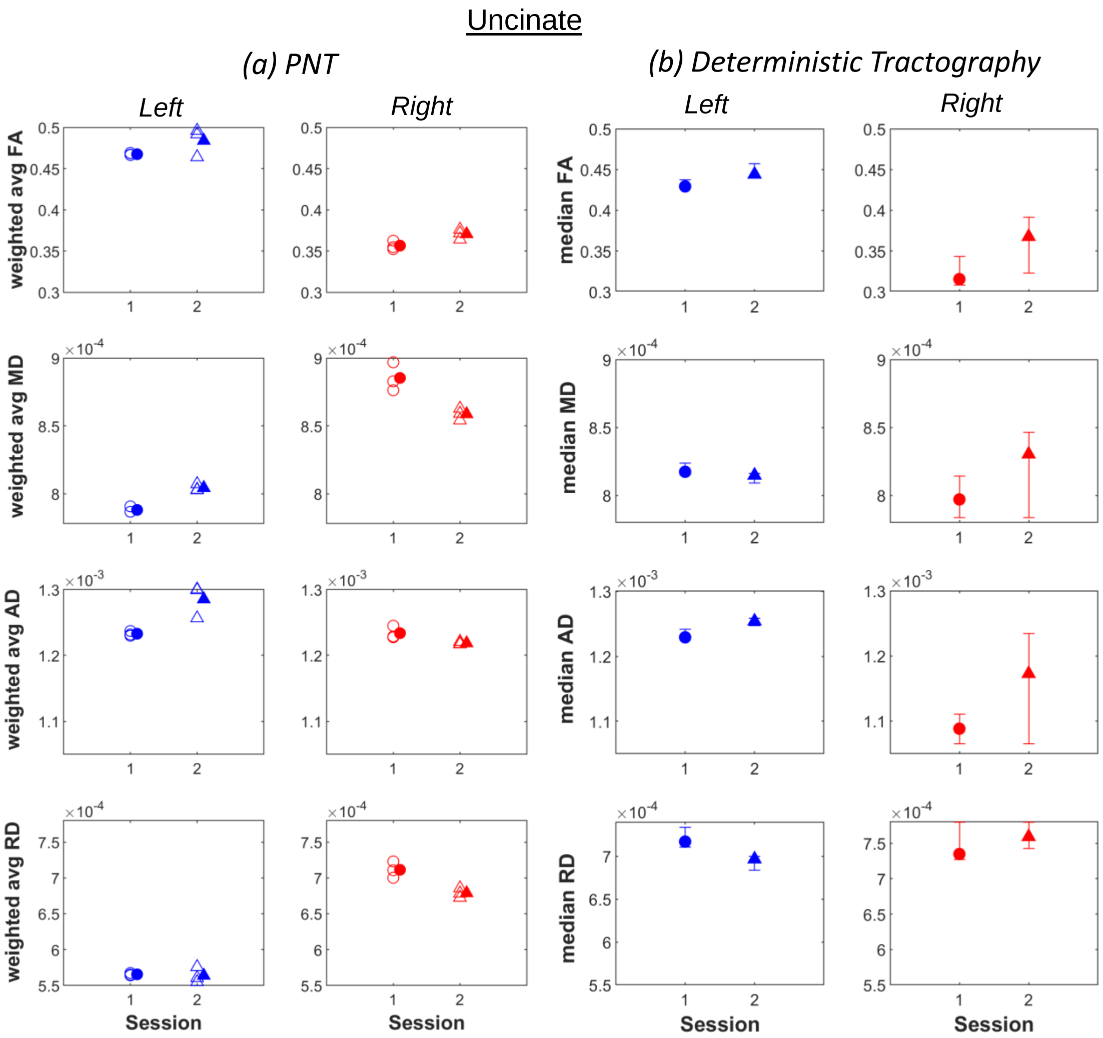

Upper panels: Best matching genu and splenium tracts segmented with PNT. Both tracts reconstructed from imaging before (session 1) and after (session 2) the piano training are presented. Lower panels: genu and splenium tracts reconstructed with deterministic tractography, also for before and after the training.

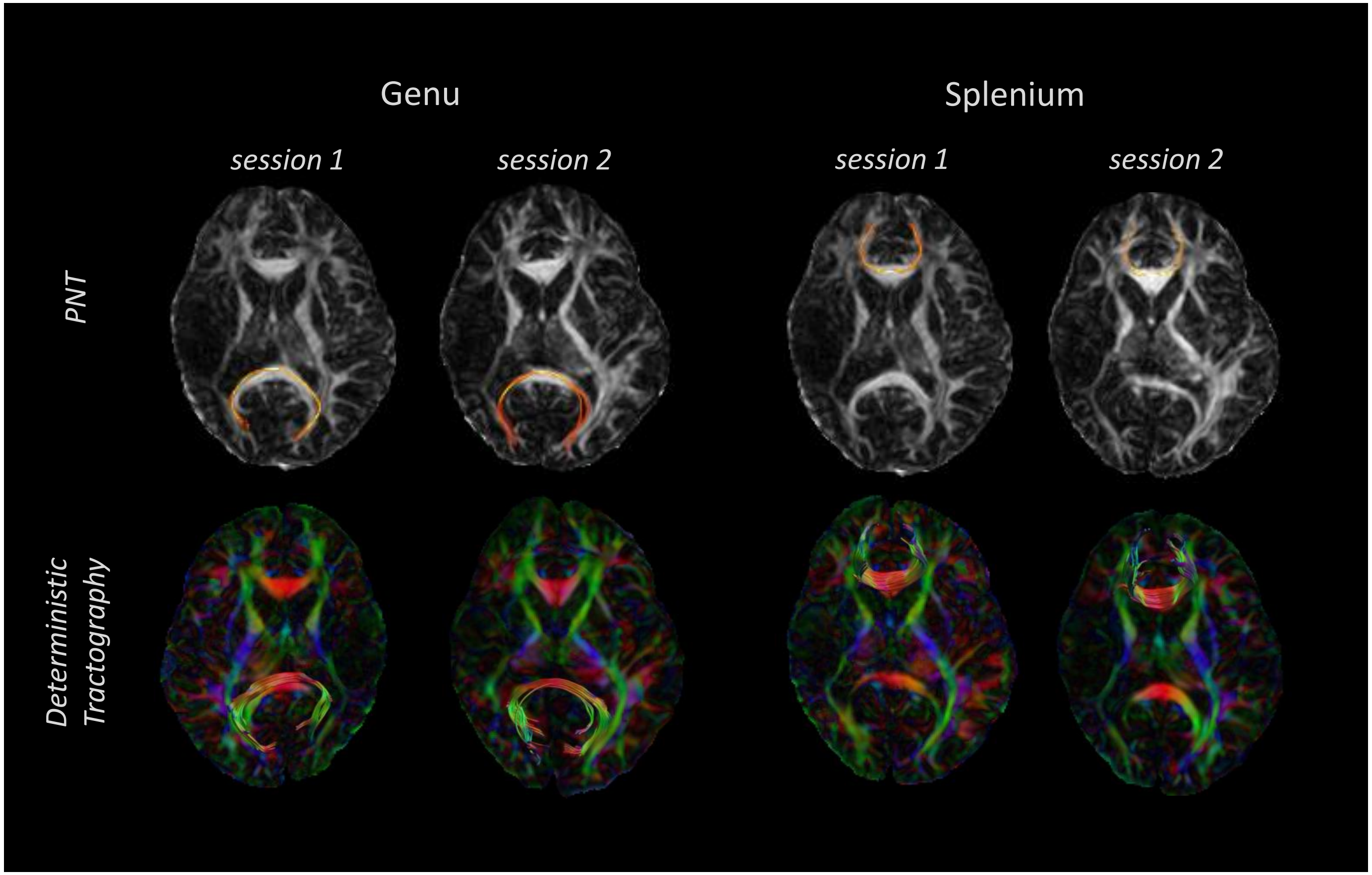

Weighted average values of FA, MD, AD and RD, computed along the best matching (relative to reference model) genu and splenium tract determined with PNT, and median values of FA, MD, AD and RD computed along the bilateral uncinate tracts derived with deterministic tractography. Error bars illustrate 95% confidence intervals. Values determined from tracts reconstructed from imaging data collected before (session 1) and after (session 2) the piano training are presented.

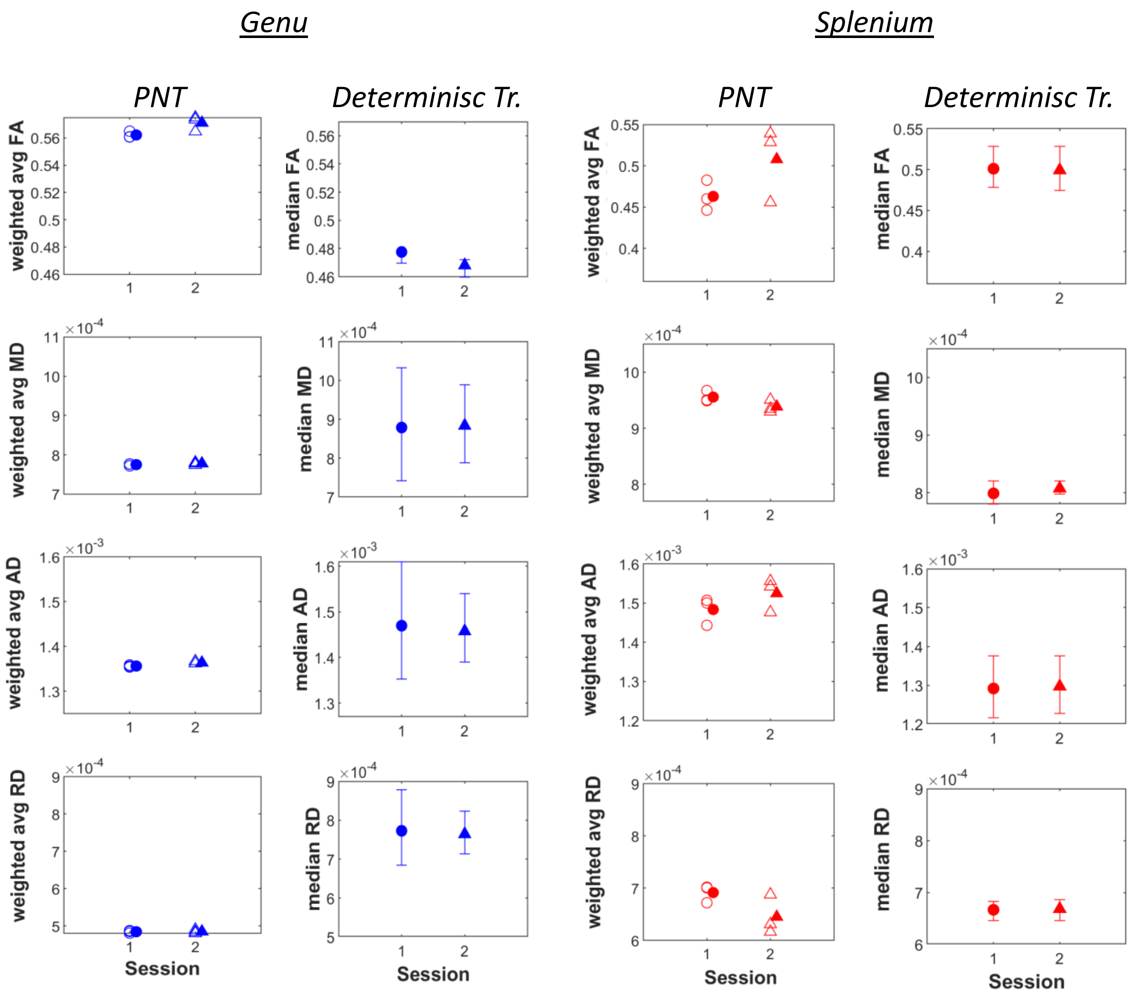

Overview of five key tracts: corticospinal tract (CST), dorsal cingulate, inferior longitudinal fasciculus, arcute and uncinate. All tracts displayed were derived from data collected after the piano training.

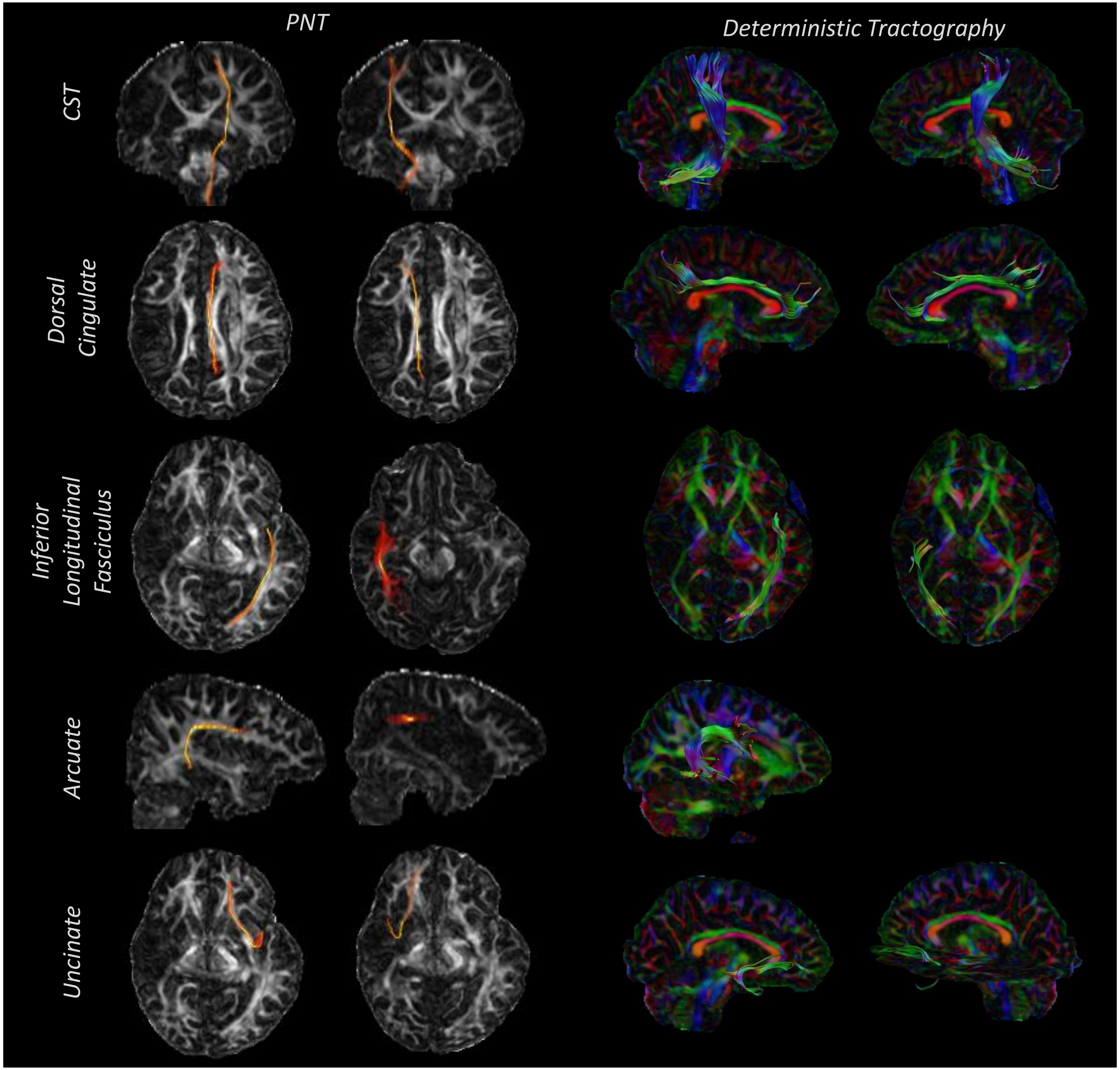

Supplement: Supplementary file 1 [file Data_Sheet_1.pdf]
